# Client satisfaction and associated factors with the health care services in Central Ethiopia, 2025: facility-based comparative cross-sectional study

**DOI:** 10.1186/s12889-026-27390-5

**Published:** 2026-04-14

**Authors:** Legese Fekede Abza, Mesfin Difer Tetema, Alemayehu Sayih Belay, Tola Getachew Bekele, Tadesse Sahle Adeba, Haimanot Abebe Geletie, Fantahun Walle Berriea, Ambaw Abebaw Emrie

**Affiliations:** 1 Department of Nursing, College of Medicine and Health Sciences, Wolkite University, Wolkite, Ethiopia; 2 Department of Midwifery, College of Medicine and Health Sciences, Wolkite University, Wolkite, Ethiopia; 3 Department of Nursing, Jimma University Medical Center, Jimma, Ethiopia

**Keywords:** Client satisfaction, Healthcare quality, Public and private hospitals, associated factors, Central Ethiopia

## Abstract

**Background:**

Client satisfaction is a key indicator of healthcare quality and the extent to which patient expectations are met. Despite efforts to improve healthcare quality, evidence on client satisfaction in public and private hospitals, particularly in Central Ethiopia, remains limited.

**Objective:**

To assess the level of client satisfaction and associated factors in public and private facilities, Central Ethiopia, 2025.

**Methods and materials:**

Facility-based comparative cross-sectional study was conducted at public and private hospitals of Central Ethiopia from April to July 2025. A systematic random sampling method was employed to select 436 participants from both institutions. A modified Client Satisfaction Assessment Tool was used to measure the level of client satisfaction. A binary logistic regression model was applied, and variables with a p-value < 0.05 with a 95% CI in the multivariable analysis were considered significant.

**Results:**

A total of 262 public and 174 private hospital clients participated. Overall satisfaction was 45%, with lower in public (39.3%) than the private (53.4%). In public hospitals, satisfaction was significantly associated with rural residence (AOR = 1.92; 95% CI: 1.09–3.37), longer duration of stay (AOR = 2.33; 95% CI: 1.15–4.72), daytime admission (AOR = 2.12; 95% CI: 1.18–3.78), and insurance payment type (AOR = 1.81; 95% CI: 1.04–3.14). In private hospitals, repeated visits (AOR = 2.44; 95% CI: 1.15–5.14), chronic illness (AOR = 2.18; 95% CI: 1.05–4.51), shorter hospital stays (AOR = 2.60; 95% CI: 1.01–6.67), and shorter waiting times (AOR = 3.43; 95% CI: 1.36–8.64) were associated. Across both facilities, attending private facilities (AOR = 1.79; 95% CI: 1.16–2.75), outpatient service use (AOR = 2.12; 95% CI: 1.15–3.93), daytime admission (AOR = 2.30; 95% CI: 1.48–3.55), and shorter hospital stays (AOR = 2.24; 95% CI: 1.33–3.79) were associated.

**Conclusion and recommendation:**

Overall client satisfaction was lower in public hospitals. Satisfaction was associated with residence, admission time, duration of stay, and payment type in public hospitals, with visit type, chronic illness, hospital stay, and waiting time in private hospitals, and with type of facility, visiting units, admission time, and duration of stay across both facilities. Improving waiting times, stay management, and payment processes is essential.

## Introduction

Client satisfaction is an indicator of the quality of care provided in medical facilities, which can be measured either directly from the patients or indirectly from the general working environment [1, 2]. It refers to the degree to which a patient feels comfortable about the service they receive from their healthcare providers in any healthcare setting [2]. It also indirectly indicates the degree of care provided and the level of communication during their stay in the healthcare facility [3].

Globally, the level of client satisfaction in public healthcare institutions varies widely, with reported estimates ranging from 60% to 95% [4–6]. Studies indicate satisfaction levels of 78%–95.2% in Saudi Arabia [4], 60.5%–97% in India [5, 6], 74% in Nepal [7], and between 50% and 65% across Sub-Saharan Africa [8, 9]. Similarly, evidence from Ethiopia shows considerable variation in client satisfaction within public healthcare institutions, with reported levels ranging from 40% to 70% [10–17].

Similarly, client satisfaction in private health facilities shows considerable variation across settings. In developed countries, reported satisfaction levels generally range from 75% to 96%, while in developing countries they vary more widely, from 35% to 90% [18–23]. In Ethiopia specifically, studies indicate that client satisfaction with private health services ranges from approximately 50% to 75% [12, 24, 25].

The level of clients’ satisfaction in public and private healthcare institutions is affected by a combination of individual, service-related, and institutional factors, and Patient-to-care giver communications [14, 17]. These include the level of the healthcare facility, clients’ socio-demographic characteristics such as age, sex, marital status, and educational status, as well as clinical and utilization-related factors including type of diagnosis, presence of chronic illness, number of visits or admissions, duration of hospital stay, and admission unit [10, 11, 23, 25]. Moreover, organizational and environmental factors; such as waiting time, type of payment, availability of prescribed drugs, accessibility of latrines, and cleanliness of consultation rooms—play a substantial role in shaping clients’ perceptions and overall satisfaction with healthcare services [7, 8, 10, 11, 14, 17, 23, 24, 26].

Client satisfaction with the healthcare service is an important determinant of healthcare quality that indicates the efficacy of services and the fulfillment of patient desires and expectations. Even though attempts to strengthen the quality of health services in Ethiopia, there is insufficient evidence regarding client satisfaction in private and public healthcare facilities, especially in Central Ethiopia. Most of the previous findings in Ethiopia have focused on either or public healthcare facilities, however few have directly compared client satisfaction across both types. Comparing the level of client satisfaction in public and private facilities can identify disparities in service quality, inform targeted interventions, and guide health policy. This is because the availability of services, provider competence, infrastructure, and cost may not necessarily be similar in private and public institutions that can significantly shape patients’ experiences and healthcare-seeking behaviors. A comparative approach is important to identify gaps in service delivery, inform targeted quality improvement strategies, and promote a patient-centered approach in both sectors. Therefore, this study was designed to assess client satisfaction and associated factors in both public and private hospitals in central Ethiopia, 2025, providing evidence to fill this knowledge gap. The findings of this study will also provide actionable insights for policymakers and health managers, supporting interventions that enhance client satisfaction, improve service utilization, and strengthen trust in the healthcare system.

## Methods and materials

### Study design and setting

Institutional-based multi-center comparative cross-sectional study was conducted among patients visiting the public and private hospitals (Butajira General Hospital and Alem General Hospital) Butajira town, East Gurage Zone, Central Ethiopia from April to July 2025.

### Inclusion and exclusion criteria

All adult clients who visited the outpatient and inpatient departments of the public and private hospitals (Butajira General Hospital and Alem General Hospital) Butajira town, East Gurage Zone, Central Ethiopia from April to July 2025. While adult patients with severe illness or critical medical conditions, cognitive impairments, or mental health problems that significantly interfere with their capacity to comprehend and engage in the research procedures were excluded were excluded.

### Sample size and sampling

The sample size was determined using Epi-info version 7.2 StatCalc with the unmatched case-control formula. Several variables has been checked to determine the sample size from previous studies and only few of the variables with optimum sample size are listed below in the Table [27]. It was determined by taking percent of controls exposed, power of 80%, ratio of 1:1, and Adjusted Odds Ratios (AOR). Cleanliness of the ward in the public hospitals of Jimma with 39.1% of controls exposed and AOR of 1.8 yield the sample size (396). Adding a 10% non-response rate, the final sample size was 436 (Table 1).

**Table 1:** Sample size determination to assess client satisfaction and associated factors with the health care services given in public and private facilities of Central Ethiopia, 2025

Variable	% of controls exposed	Power	Ratio	AOR	Sample size (*n*)
Cleanliness of the ward [27]	39.1	80	1	1.8	396
Duration of stay [27]	74.5	80	1	0.42	208
Nurses make adequate visits	29.5	80	1	1.9	360
Access to pharmacy services [27]	24.7	80	1	2.3	230
Waiting time	85.1	80	1	0.43	300

### Sampling procedure

**Fig. 1:**
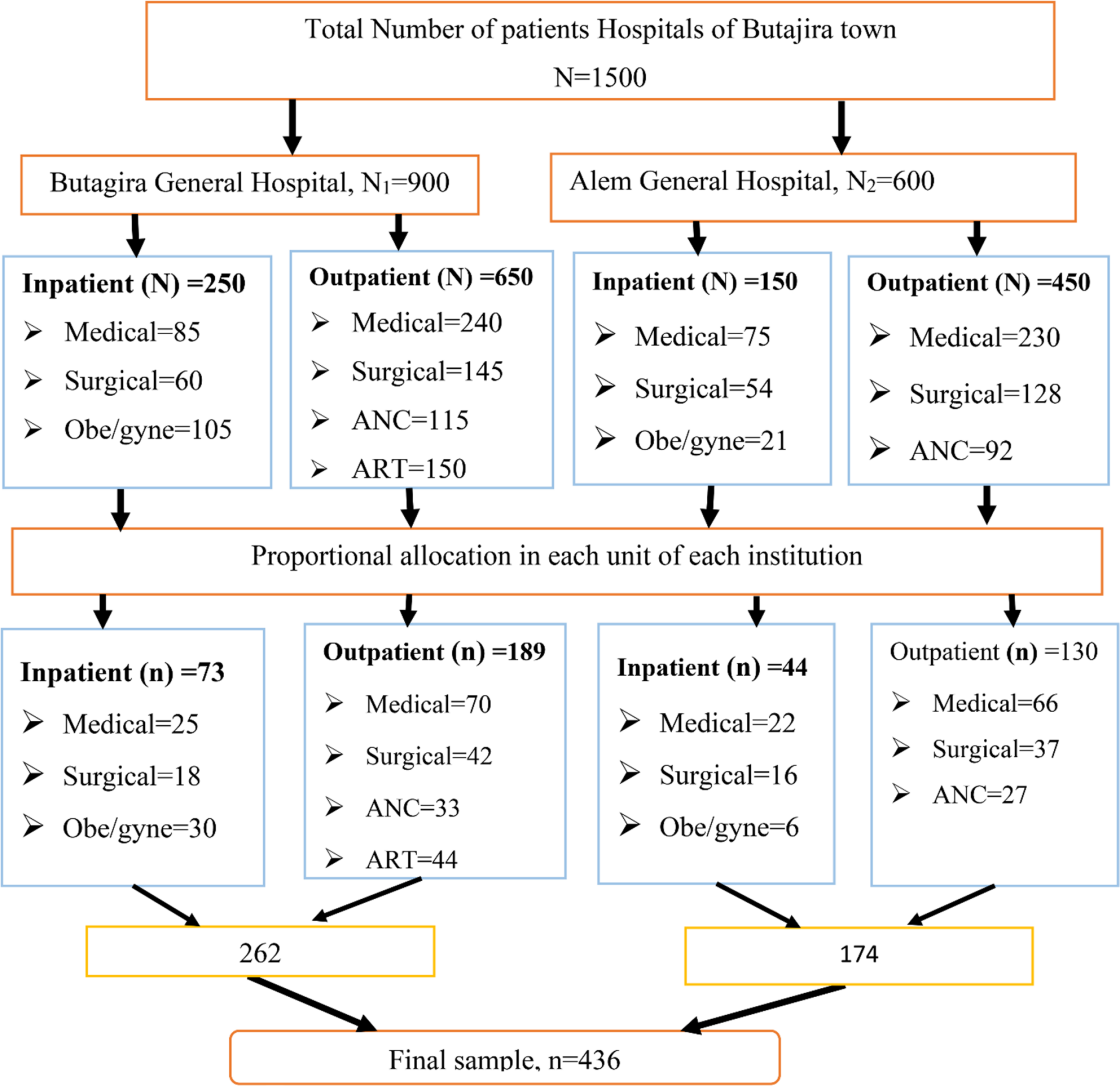
Schematic representation of sampling procedure to assess client satisfaction and associated factors with the Healthcare Services in public and private facilities of Central Ethiopia, 2025

Systematic random sampling was employed to select the study participants. All outpatient and inpatient units were included. First of all, a monthly number of clients visiting outpatient and inpatient departments of both hospitals was obtained from the registration records. Then, the number of study participants was allocated proportionally to inpatient and outpatient units of each institution. Once the allocation was determined, a sampling interval (K) was calculated for each hospital based on the monthly number of patients visiting each institution (Fig. 1). The sampling interval for each institution was calculated by the formula:

$$\:K=\frac{total\:number\:population\:}{sample\:size}$$

Based on the formula, the sampling interval for each unit of the institutions were approximately four. Finally, the first sample was selected randomly and the next patients were selected based on the interval of each institution.

### Source population

All adult clients who visited the outpatient and inpatient departments of the two hospitals.

### Study population

All adult clients who visited the outpatient and inpatient departments of the two hospitals during the data collection period.

### Variables

#### Dependent variable

I. Clients’ level of satisfaction in public healthcare facility.
II. Clients’ level of satisfaction in private healthcare facility.

#### Independent variables

Socio-demographic: age, sex, marital status, residence, occupation, and educational status.

Clinical and utilization-related: type of diagnosis, presence of chronic illness, number of visits or admissions, duration of hospital stay, type of visit, reason of visit, and admission unit.

Organizational and environmental factors: waiting time, type of payment, cost of health care services, availability of prescribed drugs, availability of laboratory service, availability of imaging tests, convenience location of OPD, accessibility of latrines, distance of hospital from home, availability of sign and directions, cleanliness of the hospital ground, cleanliness of waiting area, and cleanliness of consultation rooms.

Patient-to-care giver communications and behavior related variables: Counseling quality, provider behavior, clinical process, and privacy.

### Operational definition

Satisfied: Respondents who scored 75% or above on the satisfaction measurement scale were considered satisfied [28, 29].

Counseling quality: In this study, counseling quality was defined as availability of counseling services, time spent in counseling, clarity and comprehensiveness of information provided, each measured in a 5- point Likert scale [30].

Provider behavior: in this study provider behavior was referred to as approach (friendly/polite), respect shown, and ability to establish good rapport, each measured in a 5- point Likert scale [31].

Clinical process: the clinical process was defined as carefulness of examination, completeness of check-up, each measured in a 5- point Likert scale [31].

### Data collection tools and procedures

Data were collected using a structured interviewer-administered questionnaire developed after reviewing relevant literature on similar and related topics and adapted to the context of the current study [10, 26, 32]. The questionnaire consisted of four sections designed to collect information on socio-demographic characteristics, healthcare service–related factors, patient–caregiver communication, and clients’ satisfaction.

A modified Client Satisfaction Assessment Tool (CSAT) was used to measure clients’ satisfaction in public and private healthcare facilities that consisted of twenty (20) items, each rated on a five-point Likert scale: Very dissatisfied (1), Dissatisfied (2), Average (3), Satisfied (4), and Very satisfied (5). The responses of the 20 measuring items were summed and transformed to provide the individual level of satisfaction ranging from 1 to 100. The total possible score ranged from 20 to 100. Respondents who scored 75% or higher on the satisfaction scale were classified as satisfied. This cut-off point was used based on prior studies in similar settings, which have used ≥ 75% of the maximum possible score to categorize respondents as “satisfied” [10, 26, 32].

Data collection tools were prepared in English, translated to Amharic, retranslated to English, and checked for their consistency and clarity. A face validity was done to check the validity of the questionnaire. Moreover, the pretest was also conducted to ensure its reliability by doing the test-retest through providing of the questionnaire to the respondents twice and then the finding was confirmed for its correlation. Similarly, internal consistency was also checked to correlate the response of each question with other questions with the Cronbach alpha of 0.827. During the actual data collection, the supervisor evaluated each study site at least once per day, and anything unclear or ambiguous, and incomplete was corrected immediately.

### Data quality control

The data quality was maintained by using a carefully designed questionnaire and collected by well-trained data collectors and supervisors. Every day, the collected data were reviewed and checked for completeness and consistency by the supervisors and the principal investigator. A pretest was done on 5% of the sample size at Halaba General Hospital Ethiopia, to check the consistency of the questionnaires. The questionnaire was translated to Amharic language and then translated back into English by another expert to check for its consistency.

### Data analysis and processing

Data were collected using kobo tool box, downloaded to excel form, and exported to SPSS version 25 for analysis. Data were recoded, cleaned, and checked for the presence of potential outliers. Descriptive statistics such as frequency distribution, proportion, and percentages were done. Chi-square tests were used to determine the differences in the levels of clients’ satisfaction between the public and private healthcare facilities. Bivariable binary logistic regression analysis were used to see the association between independent and the dependent variables. Variables with a *p-value* < 0.25 were taken as candidates for the multivariable binary logistic regression to control cofounders.

The extent of multi-collinearity between independent variables was checked using variance inflation factor (VIF) and tolerance and the values of both statistics for all independent variables were within acceptable range (VIF < 10 and Tolerance > 0.1). Regression model assumptions were checked using a test like the Hosmer & Lemeshow goodness of fit with a P-value (0.183 for public, 0.885 for private, and 0.903 for overall), considered good fit for the model. Later on, multivariable binary logistic regression was done to identify factors associated with the levels of clients’ satisfaction. The association between dependent and independent variables was presented by the adjusted odds ratio (AORs) with a 95% confidence interval and a p-value of less than 0.05. Finally, results were presented in text, tables, charts, and graphs.

## Result

### Sociodemographic characteristics

A total of 262 clients from the public hospital and 174 clients from the private hospital participated in the study, yielding a 100% response rate. Out of these, 70 (26.7%) clients from the public hospital and 56 (32.2%) clients from the private hospital were under the age of 20. The majority of respondents in the public hospital were females, totaling 148 (56.5%), while in the private hospital, 94 (54.0%) of the respondents were males. Secondary education was reported by 34.7% of public-hospital respondents and 36.8% of private-hospital respondents. More than half (53.1%) of the respondents in public hospitals were urban residents and majority (47.1%) were single individuals in the private institutions (Table 2).

**Table 2:** Sociodemographic characteristics to assess client satisfaction and associated factors with the health care services in public and private facilities of Central Ethiopia, 2025

Variables	Categories	Types of hospital			
		Public		Private	
		Frequency	%	Frequency	%
Age	< 20 years	70	26.7	56	32.2
	20–29 years	62	23.7	54	31.0
	30–39 years	22	8.4	15	8.6
	40–49 years	56	21.4	18	10.3
	≥ 50 years	52	19.8	31	17.8
Gender	Male	114	43.5	94	54.0
	Female	148	56.5	80	46.0
Education	No formal education	60	22.9	24	13.8
	Primary education	57	21.8	38	21.8
	Secondary education	92	34.7	64	36.8
	College & above	54	20.6	48	27.6
Marital status	Single	106	40.5	82	47.1
	Married	135	51.5	73	42.0
	Divorced	12	4.6	7	4.0
	Widowed	9	3.4	12	6.9
Residence	Urban	139	53.1	65	37.4
	Rural	123	46.9	109	62.6

### Clinical and utilization-related

New visitors constituted 53.4% and 51.1% in the public and private hospitals, respectively. Chronic illness was more prevalent among private-hospital respondents (59.2%) than public-hospital respondents (40.8%). Approximately one-third of clients in both public (36.6%) and private hospitals (38.5%) stayed for less than 24 h, and outpatient services were the most common units visited in both public (29.4%) and private (29.9%) institutions (Table 3).

**Table 3:** Clinical and utilization related factors to assess client satisfaction and associated factors with the health care services in public and private facilities of Central Ethiopia, 2025

Variables	Categories	Types of hospital			
		Public		Private	
		Frequency	%	Frequency	%
Type of visit	New	140	53.4	89	51.1
	Repeated	122	46.6	85	48.9
Presence of chronic illness	No	155	59.2	71	40.8
	Yes	107	40.8	103	59.2
Duration of stay	< 24 h	96	36.6	67	38.5
	1–3 days	92	35.1	64	36.8
	≥ 4 days	74	28.2	43	24.7
Reason of visit	Acute illness	169	64.5	99	56.9
	Medical check up	93	35.5	75	43.1
Visiting units	Outpatient	77	29.4	52	29.9
	Follow-up/referral	74	28.2	42	24.1
	Inpatient	67	25.6	40	23.0
	Emergency	44	16.8	40	23.0
Outcome of care	Improved	126	48.1	89	51.1
	Unchanged	98	37.4	65	37.4
	Worsened	38	14.5	20	11.5
Time of admission	Day	168	64.1	108	62.1
	Night	94	35.9	66	37.9

### Organizational and environmental factors

Majority of clients received service within 15–30 min in both public (43.5%) and private (40.8%) institutions and nearly half (48.5%) of respondents in public hospitals used health insurance or received free services. In this study, most (40.2%) clients in private hospital perceived the costs of healthcare services as not affordable (Table 4).

**Table 4:** Organizational and environmental factors to assess client satisfaction and associated factors with the health care services in public and private facilities of Central Ethiopia, 2025

Variables	Categories	Types of hospital			
		Public		Private	
		Frequency	%	Frequency	%
Waiting time to get initial service	< 15 min	40	15.3	72	40.8
	15–30 min	114	43.5	52	29.9
	≥ 30 min	108	41.2	51	29.3
Type of payment	Health insurance/free	127	48.5	0	0
	Self	135	51.5	174	0.0
Cost of health care services (self-payers in public and all in private)	Affordable	44	32.6	48	27.6
	Moderately affordable	52	38.5	56	32.2
	Not affordable	39	28.9	70	40.2

### Level of client satisfactions in public and private hospitals of Butajira

**Fig. 2:**
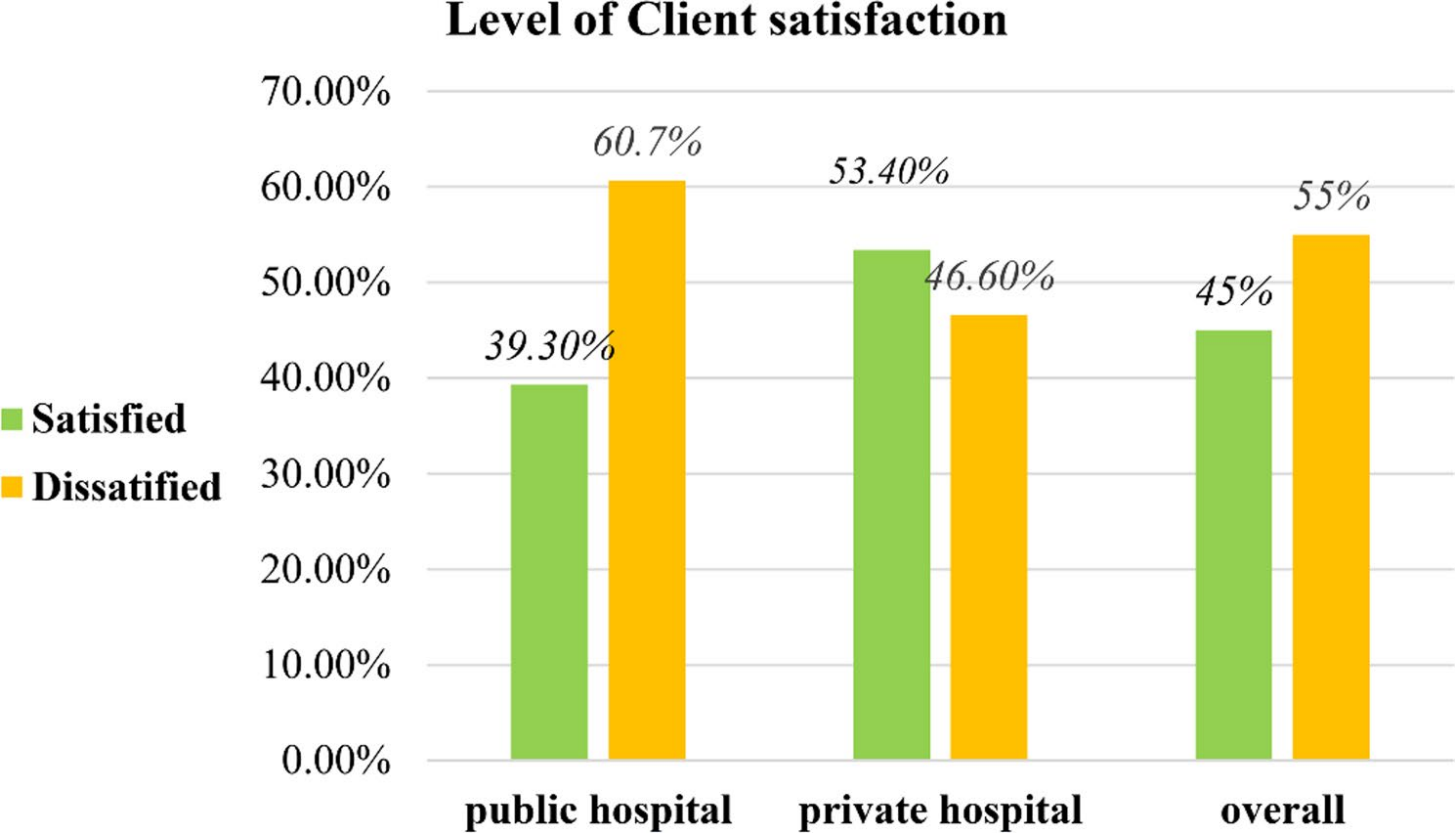
Overall level of client satisfactions with healthcare services in public and private facilities of central Ethiopia, 2025

Relatively higher satisfaction in the public hospital was observed for advice on preventing disease recurrence (85.1%), whereas lower satisfaction was reported for measures taken to assure privacy during examination (48.9%). In the private hospital, relatively higher level satisfaction was reported for the cleanliness and comfort of chairs and examination rooms (75.3%). Overall, 39.3% (with a 95% CI: 33.3, 45.3) clients in public hospital and 53.4% (with a 95% CI: 45.9, 61.2) clients in private hospital were satisfied with the overall healthcare services (Table 5). Moreover, only 45% (with a 95% CI: 39.9, 49.5) were satisfied with the healthcare services in both hospitals (Fig. 2).

**Table 5:** Level of client satisfactions with healthcare services in Public and private facilities of central Ethiopia, 2025

Items	Satisfaction s			
	Public		Private	
	0	1	0	1
	*n* (%)	*n* (%)	*n* (%)	*n* (%)
Physical facility: How much are you satisfied with…				
Heath institution compounds attractiveness	75 (28.6)	187 (71.4)	57 (32.8)	117 (67.2)
Availability of sign and directions	75 (28.6)	187 (71.4)	57 (32.8)	117 (67.2)
Suitability of the location of the institution	86 (32.8)	176 (67.2)	58 (33.3)	116 (66.7)
Cleanness and comfortability of chairs	52 (19.8)	210 (80.2)	43 (24.7)	131 (75.3)
Cleanliness of examination rooms	52 (19.8)	210 (80.2)	43 (24.7)	131 (75.3)
Accessibility & availability to health care services: How much are you satisfied with…				
Waiting time to get service after registration	74 (28.2)	188 (71.8)	57 (32.8)	117 (67.2)
Time spent for consultation	86 (32.8)	176 (67.2)	58 (33.3)	116 (66.7)
Time spent to get services & get back home	85 (32.4)	177 (67.6)	58 (33.3)	116 (66.7)
Cost paid for the services	52 (19.8)	210 (80.2)	43 (24.7)	131 (75.3)
Availability of drugs, laboratory, & imaging	84 (32.1)	178 (67.9)	58 (33.3)	116 (66.7)
Staff behavior and services: How much are you satisfied with….				
Care providers politeness and respect	81 (30.9)	181 (69.1)	58 (33.3)	116 (66.7)
Relationship with healthcare staff members	80 (30.5)	182 (69.5)	58 (33.3)	116 (66.7)
Advice on prevention recurrence of diseases	39 (14.9)	223 (85.1)	45 (25.9)	129 (74.1)
Careful examination while treating	64 (24.4)	198 (75.6)	59 (33.9)	115 (66.1)
Clarity of information provided by doctor	68 (26.0)	194 (74.0)	64 (36.8)	110 (63.2)
Information provided by nurses	59 (22,5)	203 (77.5)	48 (27.6)	126 (72.4)
The way professionals listened your health problem	56 (21.4)	206 (78.6)	50 (28.7)	124 (71.3)
Measures taken to assure privacy during examination	134 (51.1)	128 (48.9)	133 (76.4)	133 (76.4)
The opening hours of the hospital	81 (30.9)	181 (69.1)	67 (38.5)	107 (61.5)
The overall quality of health care services	61 (23.3)	201 (76.7)	50 (28.7)	124 (71.3)
Overall level of satisfaction	159 (60.7)	103 (39.3)	81 (46.6)	93 (53.4)

*Note: 0 = Dissatisfied, 1 = Satisfied

### Factors associated with client satisfaction in the public hospital

In the bivariable binary logistic regression, a total of fifteen (15) variables were included, and only six (6) variables became candidates for the multivariable binary logistic regression analysis. In the final model, four (4) variables-place of residence, duration of stay, time of admission, and type of payment become significantly associated with the level of satisfaction.

Place of residence was significantly association with the level of client satisfaction in public hospital. Compared to the urban residents, rural residents were nearly twice as likely to be satisfied with healthcare services (AOR = 1.918, with 95% CI: 1.091, 3.371). Duration of stay also showed a significant association with the level of client satisfaction in which clients who stayed less than 24 h were 2.3 times more likely to be satisfied compared to those who stayed four or more days (AOR = 2.328, with 95% CI: 1.148, 4.719).

Time of admission was another variable that demonstrated a significant association with the level of client satisfaction. The odds of being satisfied were more than two-fold among clients who visited the healthcare facility during the daytime compared to the contrary group (AOR = 2.115, with 95% CI: 1.183, 3.780). Furthermore, the type of payment for healthcare services significantly influenced the level of client satisfaction in public hospitals. Compared to self-payers, clients with Community-Based Health Insurance (CBHI) or free services were nearly twice as likely to be satisfied with healthcare services (AOR = 1.806, with 95% CI: 1.039, 3.139) (Table 6).

**Table 6:** Factors associated with client satisfaction with the healthcare services in the public facilities, Central Ethiopia, 2025

Variables	Category	Satisfaction		COR (95%CI)	AOR (95%CI)	P-value
		0	1			
Residence	Rural	73	66	2.101(1.263, 3.497)*	1.918(1.091, 3.371)*	0.024*
	Urban	86	37	1	1	
Visiting units	Outpatient	37	40	2.883(1.295, 6.416)*	2.278(0,0.967, 5.363)	0.060
	Follow-up/referral	39	35	2.393(1.070, 5.354)*	2.187(0.924, 5.179)	0.075
	Inpatient	51	16	0.837(0.351, 1.995)	0.904(0.355, 2.299)	0.832
	Emergency	32	12	1	1	
Chronic	No	84	71	1.981(1.177, 3.334)*	1.674(0.948, 2.956)	0.076
	Yes	75	32	1	1	
Duration of stay	< 24 h	51	45	2.086(1.100, 3.954)*	2.328(1.148, 4.719)*	0.019*
	1–3 days	56	36	1.519(0.792, 2.914)	1.645(0.800, 3.385)	0.176
	≥ 4 days	52	22	1	1	
Time of admission	Day	92	76	2.050(1.194, 3.519)*	2.115(1.183, 3.780)*	0.011*
	Night	67	27	1	1	
Type of payment	CBHI/free	66	61	2.047(1.236, 3.387)*	1.806(1.039, 3.139)*	0.036*
	self	93	42	1	1	

### Factors associated with client satisfaction in the private hospital

In the bivariable binary logistic regression, a total of fourteen (14) variables were included, and only seven (7) variables became candidates for the multivariable binary logistic regression analysis. In the final model, four (4) variables-type of visit, chronic illness, duration of stay, and waiting time for initial service significantly associated with the level of satisfaction.

In private hospitals, the type of visit was significantly associated with client satisfaction. Repeated visitors were over twice as likely to be satisfied with healthcare services compared to new clients (AOR = 2.435; 95% CI: 1.154–5.136). Similarly, having a chronic illness was significantly linked to higher satisfaction, with clients with chronic conditions more than twice as likely to be satisfied as those without (AOR = 2.176; 95% CI: 1.051–4.506).

Duration of stay also showed a significant association with the level of client satisfaction, in which clients who stayed less than 24 h were 2.6 times more likely to be satisfied compared to those who stayed four or more days (AOR = 2.601, with 95% CI: 1.013, 6.673). Moreover, waiting time for initial service was significantly associated with the level of satisfaction. Compared to the reference groups, clients who got initial healthcare services within less than 15 min and between 15 and 30 min were 2.7 (AOR = 2.720, with 95% 1.117, 6.624) and 3.4 (AOR = 3.427, with 95% 1.359, 8.642) times more likely to be satisfied, respectively (Table 7).

**Table 7:** Factors associated with client satisfaction with the healthcare services in the private facilities, Central Ethiopia, 2025

Variables	Category	Satisfaction		COR (95%CI)	AOR (95%CI)	P-value
		0	1			
Residence	Rural	39	26	1	1	
	Urban	42	67	2.393(1.276, 4.486)*	2.121(0.992, 4.535)	0.053
Type of visit	New	51	38	1	1	
	Repeated	30	55	2.461(1.335, 4.537)*	2.435(1.154, 5.136)*	0.019*
Chronic illness	No	57	46	1	1	
	Yes	24	47	2.427(1.297, 4.541)*	2.176(1.051, 4.506)*	0.036*
Duration of stay	< 24 h	21	46	2.767(1.252, 6.114)*	2.601(1.013, 6.673)*	0.047*
	1–3 days	36	28	0.982(0.451, 2.140)	1.096(0.439, 2.734)	0.844
	≥ 4 days	24	19	1	1	
Visiting units	Outpatient	18	34	2.556(1.094, 5.968)*	1.603(0.551, 4.664)	0.387
	Follow-up/referral	18	24	1.804(0.752, 4.329)	1.691(0.590, 4.844)	0.328
	Inpatient	22	18	1.107(0.457, 2.679)	0.551(0.179, 1.700)	0.300
	Emergency	23	17	1	1	
Time of admission	Day	42	66	2.270(1.215, 4.240)*	1.900(0.901, 4.003)	0.092
	Night	39	27	1	1	
Waiting time to initial service	< 15 min	28	43	3.359(1.572, 7.177)*	2.720(1.117, 6.624)*	0.028*
	15–30 min	18	34	4.132(1.816, 9.404)*	3.427(1.359, 8.642)*	0.009*
	≥ 30 min	35	16	1	1	

### Factors associated with client satisfaction in both the public and private facilities (combined model)

In the bivariable binary logistic regression, a total of fourteen (15) variables were included, and only five (5) variables became candidates for the multivariable binary logistic regression analysis. In the final model, four (4) variables-type of facility, visiting units, time of admission, and duration of stay significantly associated with the level of satisfaction.

The type of facility was significantly associated with client satisfaction, with clients visiting private facilities being 1.786 times more likely to be satisfied with healthcare services (AOR = 1.786; 95% CI: 1.159–2.752). Similarly, the visiting unit significantly influenced satisfaction in both public and private hospitals. Clients attending outpatient units (AOR = 2.123; 95% CI: 1.147–3.930) or follow-up/referral units (AOR = 1.975; 95% CI: 1.063–3.669) were nearly twice as likely to report satisfaction with healthcare services.

Time of admission was another variable that demonstrated a significant association with the level of client satisfaction in both facilities. The odds of being satisfied were more than two-fold among clients who visited the healthcare facility during the daytime compared to the contrary group (AOR = 2.300, with 95% CI: 1.488, 3.556). Duration of stay also showed a significant association with the level of client satisfaction, in which clients who stayed less than 24 h were 2.2 times more likely to be satisfied compared to those who stayed four or more days (AOR = 2.242, with 95% CI: 1.325, 3.793) (Table 8).

**Table 8:** Factors associated with client satisfaction in the public and private facilities, Central Ethiopia, 2025

Variables	Category	Satisfaction		COR (95%CI)	AOR (95%CI)	P-value
		0	1			
Type of institution	Public	159	103	1	1	
	Private	81	93	1.772(1.203, 2.611)*	1.786(1.159, 2.752)*	0.009*
visiting units	Outpatient	55	74	2.552(1.444, 4.509)*	2.123(1.147, 3.930)*	0.017*
	Follow-up/referral	57	59	1.963(1.101, 3.501)*	1.975(1.063, 3.669)*	0.031*
	Inpatient	73	34	0.883(0.482, 1.620)	0.733(0.382, 1.407)	0.350
	Emergency	55	29	1	1	
Admission time	Day	134	142	2.080(1.389, 3.115)*	2.300(1.488, 3.556)*	0.000*
	Night	106	54	1	1	
Duration of stay	< 24 h	72	91	2.343(1.436, 3.823)*	2.242(1.325, 3.793)*	0.003*
	1–3 days	92	64	1.290(0.785, 2.118)	1.310(0.770, 2.229)	0.320
	≥ 4 days	76	41	1	1	
Waiting time initial service	< 15 min	49	62	2.264(1.380, 3.716)*	1.613(0.933, 2.790)	0.087
	15–30 min	89	77	1.548(0.992, 2.416)	1.304(0.805, 2.112)	0.281
	≥ 30 min	102	57		1	

## Discussion

This study aimed to determine the level of client satisfaction and factors associated with it with the healthcare services in public and private health institutions of Butajira town, Central Ethiopia. The current study indicated that 39.3% (with a 95% CI: 33.3, 45.3) clients in public hospital were satisfied with the overall healthcare services. It is in line with studies conducted in North Shoa of Ethiopia (44.6%) [33], Tigray (41.4%) [34], West Amhara region (39.3%) [35].

It is lower when compared to studies conducted in Bangladesh (51%) [36], Nepal (61.8%) [37], Rwanda (81.6%) [38], Mekele (72%) [26], Hawassa (72.9%) [39], Jimma (54.2%) [12], and Addis Ababa (77.4%) [40]. However, it higher when compared to the findings of the studies carried out in Jimma (27.8%) [41]. Differences in patient characteristics, measurement tools, and study settings may account for the observed variation in client satisfaction levels. Furthermore, differences in infrastructure, availability of resources, patient expectations, and sociodemographic factors significantly influence overall satisfaction [16, 25].

This study revealed that 53.4% (with a 95% CI: 45.9, 61.2) clients in private hospital were satisfied with the overall healthcare services. It is consistent with the studies conducted in Jimma (57.1%) [12]. The finding was lower compared to the studies carried out in Bangladesh (75%) [36] and Hawassa (80.3%) [39]. However, the current finding was higher compared to a study conducted in Nepal (37.27%) [37]. The observed discrepancy in the level of client satisfaction in private healthcare facilities could be attributed to the difference in quality of services, resource availability, and infrastructures. The type of services utilized, patient prospects, and patient-to-healthcare provider communication may also play an important role in the level of satisfaction. Variations in tools, cut-off points, data collection periods, and costs of services could also bring inconsistencies in the level of satisfaction across studies [25, 42–44].

Overall, only 45% (with a 95% CI: 39.9, 49.5) of clients were satisfied with the healthcare services in both hospitals. This finding is in line with studies conducted in Nepal [37] and Jimma (46.2%) [45]. However, the current finding was lower compared to the studies carried out in Bangladesh (65%) [36] and East Ethiopia (65.6%) [46]. The variation in overall satisfaction levels across studies may be due to differences in service quality, the timing of surveys, sociodemographic characteristics, measurement tools (as this study employed a 20-point scale questionnaire), regional and cultural factors, and health system reforms.

The results of this study indicated that private hospitals had greater levels of patient satisfaction than public hospitals. This finding is consistent with the studies carried out in India [47], Bahir Dar [24], Hawassa [39], and Jimma [12] in which the level of client satisfaction was higher among private hospitals compared to the public ones. However, the finding of this study was inconsistent with studies conducted in Nepal [37]. The inconsistency could be attributed to service affordability, the complexity of cases, and measuring tools. In certain circumstances, clients in public health institutions may have higher levels of satisfaction due to several reasons, like the costs of services, proper institutional support, or lower client expectations. Conversely, clients might have higher expectations from private institutions that may not always necessarily be met [44, 48, 49].

The current study showed that rural residence was significantly associated with higher client satisfaction in public hospitals compared to urban residence. This could be due to relatively lower expectations and limited engagement to alternative healthcare services. Cultural norms and courtesy bias, in which patients may provide favorable response out of politeness rather than the actual opinions, may increase the rate of satisfaction among rural residents. Moreover, adaptation to resource-limited healthcare settings may contribute to relatively higher level of satisfaction among rural residents. These findings are supported by similar evidence from Ethiopia and other low-income settings, where satisfaction reflects not only service quality but also expectations and sociocultural contexts [50, 51].

Duration of stay also showed a significant association with the level of client satisfaction across public, private, and combined analyses, in which those who stayed less than one day had relatively higher level of satisfaction compared to patients who stayed four days or longer. This might be explained by patient expectations, in which patients whose care is provided within a short period of time are more likely to appreciate that their needs have been met, which is associated with relatively higher level of satisfaction. Shorter stay is often considered efficient and effective, whereas longer stay may result in poor communication and increase facility-related stressors. Furthermore, shorter stay reduces financial burden and hospital-related complications, and it is often associated with better perceive outcomes and relatively higher level of satisfaction [12, 52–54].

Additionally, the mode of payment significantly affected client satisfaction in public hospitals, with clients using CBHI or receiving free services nearly twice as likely to be satisfied compared to self-paying patients. This could be due to there is a reduced financial burden and lowered stress regarding payment which may enhance the overall positive perception towards the care provided and adherence to recommended services subsequently result in higher satisfaction compared to the self-paying counter-parts [46, 55].

In private hospitals, the type of visit significantly influenced client satisfaction. Clients who returned for repeat visits were more than twice as likely to report satisfaction compared to those visiting for the first time. This could be explained by the fact that clients with repeated visits to the healthcare facilities become more familiar with the overall system of the hospital, establish relationships with the healthcare providers, and have better continuity of care [56]. Moreover, clients having prior positive experiences helps align expectations that in turn enhance subsequent visits, making them more likely to meet and further improve client satisfaction [56–58].

Having a chronic illness was significantly associated with higher client satisfaction, with patients living with chronic conditions being more than twice as likely to report satisfaction compared to those without such conditions. This could be attributed to the fact that patients with chronic comorbid conditions often have long-term engagement with the healthcare services that strongly enhances a good continuity of care and patient-to-provider relation that further enhance the overall care experience. Clients with chronic conditions often have frequent interaction with the health system, which help them to be familiar with the providers and increased for self-management with various lifestyle mechanisms, enhancing confidence and perceived quality of care. In private hospitals, personalized attention and coordinated services further reinforce positive experiences that subsequently improve the level of satisfaction with the overall healthcare services [54, 59, 60].

The waiting time for initial service was significantly linked to the level of satisfaction. In comparison to the reference groups, clients who received initial healthcare services in less than 15 min and those who waited between 15 and 30 min were nearly three times more likely to express satisfaction. Shorter waiting times for initial service improve patient satisfaction by meeting patients’ expectations for timely care and reducing anxiety and frustration. Studies in Ethiopian public hospitals have consistently shown that patients who receive prompt services report higher satisfaction compared to those who experience longer waits [61, 62]. On the other hand, the study showed slightly higher odds of satisfaction for the 15–30 min waiting time group compared to the < 15 min group. This is a bit confusing, but this could be explained by patients’ perceived quality of care and expectations, in which patients waiting less than 15 min may perceive the healthcare services as rushed or superficial; in contrast, a moderate waiting time (15–30 min) may enable patients to feel that providers are spending relatively adequate time with each patient, resulting in higher perceived level of satisfaction [63].

The study found that clients attending private hospitals were almost twice as likely to report satisfaction with healthcare services compared to those visiting public hospitals. This could be likely due to the greater ability of private facilities to provide timely and client-centered care, including shorter waiting times, longer consultation periods, and more attentive patient-provider interactions, which might be associated with the fulfillment of perceived patient expectation. In contrast, public institutions with resource-limited set-ups often face higher patient flows, which may limit consultation time and increase waiting time that could be associated with a negative patient experience and comparably lower level of satisfaction [25, 64].

The hospital unit visited significantly influenced client satisfaction in both public and private hospitals, with those attending outpatient and follow-up/referral units being nearly twice as likely to report satisfaction with healthcare services. This could be due to differences in service delivery systems and expectations of patients across admission units. Outpatient and follow-up/referral units often characterized by organized consultations, communication of plans of care that in turn improve the level of client satisfaction [10, 65, 66].

The time of admission was significantly associated with client satisfaction across public institution and combined analysis (public and private together), with patients admitted during the daytime having a higher tendency of being satisfied compared to those admitted at night. This may be attributed to greater staff availability, better supervision, and shorter waiting times for services during daytime hours. Conversely, nighttime admissions are often associated with reduced staffing, delayed diagnosis, and limited access to services, which may result in lower satisfaction, especially in resource-limited facilities. These findings are consistent with evidence from previous studies [63, 67].

### Limitations of the study

Since the study was cross-sectional design, it may limit the ability to establish causal relationships between identified factors and client satisfaction. The relatively small and uneven sample size, along with reliance on self-reported data, may introduce selection, recall, and social desirability biases. Additionally, the study focused on specific sociodemographic and service-related factors, and findings from the selected public and private hospitals may not be generalizable to other settings or regions.

## Conclusion

This study suggests that overall client satisfaction was relatively lower in public hospitals compared to private hospitals in Addis Ababa. In public hospitals, factors such as place of residence, duration of hospital stay, time of admission, and type of payment were significantly associated with client satisfaction. In private hospitals, type of visit (new or follow-up), presence of chronic illness, duration of hospital stay, and waiting time for initial service were associated with satisfaction. Across both settings, type of facility, visiting units, time of admission, and duration of stay appeared to be important factors. However, due to the cross-sectional design, causal relationships cannot be established, and the findings should be interpreted with caution. Public hospitals should prioritize reducing waiting time at registration and initial consultation, improving patient flow and discharge processes, and simplifying payment procedures. Special attention is needed during peak admission times through better staff allocation. Private hospitals should focus on reducing initial waiting time and strengthening follow-up care, particularly for patients with chronic illnesses. In both settings, improving service organization within visiting units and facility conditions is recommended. Future studies should include larger samples and assess additional factors such as provider communication and patient expectations.

## Data Availability

The datasets created and analyzed during the current study are available from the corresponding author upon a reasonable request.

## Abbreviations

AORs: Adjusted Odds Ratios
CBHI: Community–Based Health Insurance
CI: Confidence Interval
COR: Crude Odds Ratio
CSAT: Client Satisfaction Assessment Tool
OPD: Outpatient Department
SPSS: Statistical Package for the Social Sciences
VIF: Variance Inflation Factor
